# Dissociation of Fatty Liver and Insulin Resistance in I148M PNPLA3 Carriers: Differences in Diacylglycerol (DAG) FA18:1 Lipid Species as a Possible Explanation

**DOI:** 10.3390/nu10091314

**Published:** 2018-09-17

**Authors:** Andras Franko, Dietrich Merkel, Marketa Kovarova, Miriam Hoene, Benjamin A. Jaghutriz, Martin Heni, Alfred Königsrainer, Cyrus Papan, Stefan Lehr, Hans-Ulrich Häring, Andreas Peter

**Affiliations:** 1 Department of Internal Medicine IV, Division of Endocrinology, Diabetology, Angiology, Nephrology and Clinical Chemistry, University Hospital Tübingen, 72076 Tübingen, Germany; kovarova-marketa@seznam.cz (M.K.); miriam.hoene@med.uni-tuebingen.de (M.H.); benjamin.jaghutriz@med.uni-tuebingen.de (B.A.J.); martin.heni@med.uni-tuebingen.de (M.H.); hans-ulrich.haering@med.uni-tuebingen.de (H.-U.H.); andreas.peter@med.uni-tuebingen.de (A.P.); 2 Institute for Diabetes Research and Metabolic Diseases of the Helmholtz Center Munich, University of Tübingen, 72076 Tübingen, Germany; 3 German Center for Diabetes Research (DZD e.V.), 85764 Neuherberg, Germany; stefan.lehr@ddz.uni-duesseldorf.de; 4 Sciex Germany GmbH, 64293 Darmstadt, Germany; dietrich.merkel@sciex.com (D.M.); cyrus.papan@sciex.com (C.P.); 5 Department of General, Visceral and Transplant Surgery, University Hospital Tübingen, 72076 Tübingen, Germany; alfred.koenigsrainer@med.uni-tuebingen.de; 6 Institute for Clinical Biochemistry and Pathobiochemistry of DDZ, University of Düsseldorf, 40225 Düsseldorf, Germany

**Keywords:** NAFLD, liver, PNPLA3, diacylglycerol, lipidomics

## Abstract

Fatty liver is tightly associated with insulin resistance and the development of type 2 diabetes. I148M variant in patatin-like phospholipase domain-containing protein 3 (PNPLA3) gene is associated with high liver fat but normal insulin sensitivity. The underlying mechanism of the disassociation between high liver fat but normal insulin sensitivity remains obscure. We investigated the effect of I148M variant on hepatic lipidome of subjects with or without fatty liver, using the Lipidyzer method. Liver samples of four groups of subjects consisting of normal liver fat with wild-type PNPLA3 allele (group 1); normal liver fat with variant PNPLA3 allele (group 2); high liver fat with wild-type PNPLA3 allele (group 3); high liver fat with variant PNPLA3 allele (group 4); were analyzed. When high liver fat to normal liver fat groups were compared, wild-type carriers (group 3 vs. group 1) showed similar lipid changes compared to I148M PNPLA3 carriers (group 4 vs. group 2). On the other hand, in wild-type carriers, increased liver fat significantly elevated the proportion of specific DAGs (diacylglycerols), mostly DAG (FA18:1) which, however, remained unchanged in I148M PNPLA3 carriers. Since DAG (FA18:1) has been implicated in hepatic insulin resistance, the unaltered proportion of DAG (FA18:1) in I148M PNPLA3 carriers with fatty liver may explain the normal insulin sensitivity in these subjects.

## 1. Introduction

Nonalcoholic fatty liver disease (NAFLD) is characterized by elevated hepatic lipid content [1]. NAFLD is claimed to be a benign illness, however, it can further develop to nonalcoholic steatohepatitis (NASH), liver fibrosis, cirrhosis and hepatocellular carcinoma [2]. The prevalence of NAFLD is continuously increasing, and currently it is estimated to be higher than 20% in industrialized countries [3]. Despite this high prevalence of NAFLD and its complication NASH, there is no established effective drug therapy, which is generally approved for these illnesses [4]. NAFLD/NASH patients are advised to lose weight with lifestyle intervention (mostly consisting of healthy diet and exercise [4]), however, not all patients benefit from these interventions [5]. To treat NAFLD/NASH, several drugs have been shown to be beneficial in animal models [6,7], and novel activators for peroxisome proliferator-activated receptor (PPAR) (elafibranor) and farnesoid X receptor (FXR) (obeticholic acid) are currently under phase 3 studies in human cohorts with promising results [4]. NAFLD is a strong determinant of insulin sensitivity and the development of type 2 diabetes, however, some distinct genetic causes for the dissociation of liver fat content and insulin sensitivity have been identified [1].

The rs738409 C>G single nucleotide polymorphism (SNP) in the patatin-like phospholipase domain-containing protein 3 (PNPLA3) gene is a common inherited trait, which results in an amino acid exchange I148M leading to a functional mutation of PNPLA3 [8]. At this position, both hetero- (I148M) and homozygous (M148M) variants are described. The prevalence of PNPLA3 variants vary due to ethnicity of the population [9] and 34–37% (I148M) and 4–9% (M148M) are described in studies analyzing German or European-American populations, respectively [8,10]. Several studies demonstrated that I148M PNPLA3 carriers showed an altered metabolic phenotype on nutritional challenges compared to wild-type subjects. Dietary intake of carbohydrates was shown to modify the association between PNPLA3 genotype and circulating triglyceride levels [11]. Carbohydrate overfeeding led to an increased de-novo lipogenesis in proportion to the increase in liver fat and serum triglycerides in subjects with I148I carriers, which however, was not observed in M148M carriers [12]. Furthermore, the M148M PNPLA3 variant influenced the changes in liver fat and docosahexaenoic acid tissue enrichment during a 15–18 months addition of omega 3 fatty acids [13]. The presence of rs738409 SNP was positively associated with elevated liver fat, however, carriers do not show insulin resistance [8,10,14]. On the other hand, some subjects carrying the I148M variant show normal liver fat content, and the function of PNPLA3 in these subjects is not studied yet. PNPLA3 has several enzyme activities, it was reported to be involved in lipid hydrolysis (as a triacylglycerol lipase) and synthesis (as a lysophosphatidic acyltransferase) [15]. The I148M variant is claimed to show lower lipolysis of hepatic triacylglycerols (TAGs) [16] and elevated hepatic TAG synthesis [17]. However, for subjects carrying I148M PNPLA3 variant a lower de novo lipogenesis is also reported [18]. The discrepancy between high liver TAG level and normal insulin sensitivity in I148M carriers is not resolved yet [19], but altered hepatic TAG pattern, especially long-chain polyunsaturated fatty acid content, is reported in rodent and human studies analyzing I148M PNPLA3 carriers [14,15,20]. Several lipid species are implicated in the state of insulin resistance in patients with NAFLD [21]. Elevated ceramide [15] or high diacylglycerol (DAG) content [22,23] have been found in the liver of rodents and human subjects with insulin resistance. Furthermore, quantitative measurements of unique lipid species have been previously shown to broaden our knowledge to understand complex diseases, such as cystic fibrosis, NAFLD and type 1 diabetes and pave the way for identifying new lipid biomarkers [24,25,26,27].

In order (i) to study the function of I148M PNPLA3 variant in subjects with normal and high liver fat and, (ii) to analyze the key metabolic lipids (also including detailed measurements of individual ceramide and diacylglycerol lipid species) possibly evoking insulin resistance in wild-type but not in I148M PNPLA3 carriers, we studied the liver of subjects with normal and high hepatic TAG content with wild-type or I148M (heterozygous), as well as M148M (homozygous) PNPLA3 variants. To examine the lipid profiles, we decided to perform an unbiased lipidomic analysis using the Lipidyzer platform, which was originally established for plasma samples [28], therefore, we applied it with an adapted protocol for solid tissue lysates.

## 2. Materials and Methods

### 2.1. Human Liver Samples, Total Liver TAG Measurement, and PNPLA3 Genotyping

For the analysis of liver tissue samples, a cohort of European descendent men and women undergoing liver surgery at the Department of General, Visceral, and Transplant Surgery at the University Hospital of Tübingen was included in the present study. None of the patients were diagnosed with an abuse of alcohol, however, no detailed data on alcohol consumption was consistently collected. The liver tissue was collected during hepatic surgery that was performed for different reasons, e.g., hepatic hemangioma, curative resection of hepatic metastases of colorectal malignancies or hepatocellular carcinoma. Patients fasted overnight before collection of liver samples. Exclusion criteria were viral hepatitis infection and liver cirrhosis. Informed, written consent was obtained from all participants, and the Ethics Committee of the University of Tübingen approved the protocol (239/2013BO1) according to the Declaration of Helsinki. Liver samples taken from normal, non-diseased tissue, were quickly frozen in liquid nitrogen and stored at −80 °C. To measure total TAG content, liver tissue samples were homogenized in phosphate buffered saline containing 1% Triton X-100 with a TissueLyser (Qiagen, Hilden, Germany) and determined as described previously [29,30]. In order to match subjects for similar body weight, BMI (body mass index) and age as well as for different liver TAG content, subjects showing less than 3.0% liver TAG content were classified as normal TAG group and subjects showing more than 4.3% liver TAG were classified as high TAG group. For PNPLA3 genotyping, total DNA was isolated from whole blood using a DNA isolation kit (NucleoSpin, Macherey and Nagel, Düren, Germany). The I148M PNPLA3 variations were genotyped using Sequenom’s massARRAY System with iPLEX software (Sequenom, Hamburg, Germany) as described previously [14]. Plasma ALT levels were measured with routine clinical chemistry [10].

### 2.2. Lipidyzer Platform

The Lipidyzer™ platform (SCIEX, Darmstadt, Germany) was used for the whole lipid analysis work flow. Briefly, 10 mg liver was solubilized in 100 µL internal standards (IS, Avanti Polar Lipids, Inc., AL, USA) and 200 µL of 75% methanol was added and hepatic lipids were extracted using methyl tert-butyl ether (MTBE) as described previously [31]. The following isotopes labeled internal standards were used dCER(d16:0), dCE(16:0), dCE(16:1), dCE(18:1), dCE(18:2), dCE(20:3), dCE(20:4), dCE(20:5), dCE(22:6), dDAG(16:0/16:0), dDAG(16:0/18:0), dDAG(16:0/18:1), dDAG(16:0/18:2), dDAG(16:0/18:3), dDAG(16:0/20:4), dDAG(16:0/20:5), dDAG(16:0/22:6), dFFA(16:0), dFFA(17:1), dLPC(16:0), dLPE(18:0), dPC(16:0/16:1), dPC(16:0/18:1), dPC(16:0/18:2), dPC(16:0/18:3), dPC(16:0/20:3), dPC(16:0/20:4), dPC(16:0/20:5), dPC(16:0/22:4), dPC(16:0/22:5), dPC(16:0/22:6), dPE(18:0/18:1), dPE(18:0/18:2), dPE(18:0/18:3), dPE(18:0/20:3), dPE(18:0/20:4), dPE(18:0/20:5), dPE(18:0/22:5), dPE(18:0/22:6), dSM(16:0), dSM(18:1), dSM(24:0), dSM(24:1), dTAG50:1-FA16:0, dTAG52:1-FA18:0, dTAG52:2-FA18:1, dTAG52:3-FA18:2, dTAG52:4-FA18:3, dTAG54:4-FA20:3, dTAG54:5-FA20:4, dTAG56:7-FA22:6, dDCER(16:0), dHCER(16:0), and dLCER(16:0). For each injection, 50 µL of extracted lipid sample was introduced by flow injection (FIA) using a Nexera X2 system (Shimadzu Germany GmbH, Duisburg, Germany), equipped with a 50 µL-sample loop. A Lipidyzer™ included 750 × 0.05 mm PEEKsil™ (Trajan Scientific Europe Ltd., Milton Keynes, UK) sample tubing was used to connect the autosampler valve with the grounding union on the electrospray ionization (ESI) source, and a 350 × 0.05 mm PEEKsil™ (Trajan Scientific Europe Ltd, Milton Keynes, UK) sample tubing was used to connect the grounding union with the ESI electrode having 65 µm inner diameter. The flow profile for the flow injection was determined by the Lipidyzer™ acquisition method with a flow rate during the data acquisition period being 7 µL/min. The mass spectrometry analysis was performed on a Lipidyzer™ Platform, including the Sciex QTRAP^®^ (SCIEX, Darmstadt, Germany) 5500 system equipped with SelexION^®^ (SCIEX, Darmstadt, Germany) Technology (differential mobility separation, DMS). Multiple reaction monitoring (MRM) was used to target and quantitate several hundreds of lipid molecular species from 13 different lipid classes. All samples were first measured in positive and negative polarity with SelexION separation, followed by measurement without SelexION separation. The acquisition time per sample took approximately 25 min for the complete acquisition. All data was acquired and processed automatically using the Lipid Manager Workflow software (SCIEX, Darmstadt, Germany). This provides the following data tables: (i) quantitative results for each lipid class as a sum of individual species; (ii) mole percent composition obtained computationally from lipid molecular species data; and (iii) accurate lipid species compositions.

### 2.3. Data Evaluation

Missing values, which were not possible to measure and showed zero values, were handled as follows: From the complete data set, lipids, which showed higher values than zero at least in 50% of any group were kept, otherwise they were discarded. To determine whether the groups separate from each other according to PNPLA3 genotype or hepatic TAG content, multivariate partial least squares discriminant analysis (PLS-DA) were performed using soft independent modeling of class analogy (SIMCA, Umetrics, Umea, Sweden). The 761 individual lipid species and the 84 sum of lipid classes were summed up and these 845 lipid values were logarithmic transformed and were statistically evaluated, as written below.

### 2.4. Statistics

To determine statistical different lipid species caused by increased liver fat, group 3 (high liver fat, wild-type PNPLA3 allele) was compared to group 1 (normal liver fat, wild-type PNPLA3 allele) as well as group 4 (high liver fat, variant PNPLA3 allele) was compared to group 2 (normal liver fat, variant PNPLA3 allele) using GraphPad Prism (7.03). Multiple t-tests were applied with Benjamini-Hochberg correction and false discovery rate (FDR) was set <5%. Furthermore, analysis of variance (ANOVA) with Holm-Sidak´s post hoc test were applied as it is indicated.

## 3. Results

### 3.1. Characteristics of Study Groups

To distinguish between the effect of increased liver TAG content and PNPLA3 genotype, subjects were matched for body weight, BMI and age and divided into the following four groups: subjects showing normal liver fat with wild-type PNPLA3 (group 1); normal liver fat with I148M PNPLA3 variant (group 2); high liver fat with wild-type PNPLA3 (group 3) and high liver fat with I148M PNPLA3 variant (group 4) (Table 1).

Our study groups consisted of overweight subjects with similar age, weight, BMI and ALT levels (Table 1). Liver fat was significantly higher in high TAG groups compared to normal groups (group 3 vs. 1 and group 4 vs. 2), but was not different in I148M PNPLA3 carriers compared to wild-type carriers (Table 1). Both variant PNPLA3 groups (groups 2 and 4) consisted of one homozygous M148M (MM) carrier and the rest of the subjects were heterozygous I148M (IM) carriers (Table 1).

**Figure 1:**
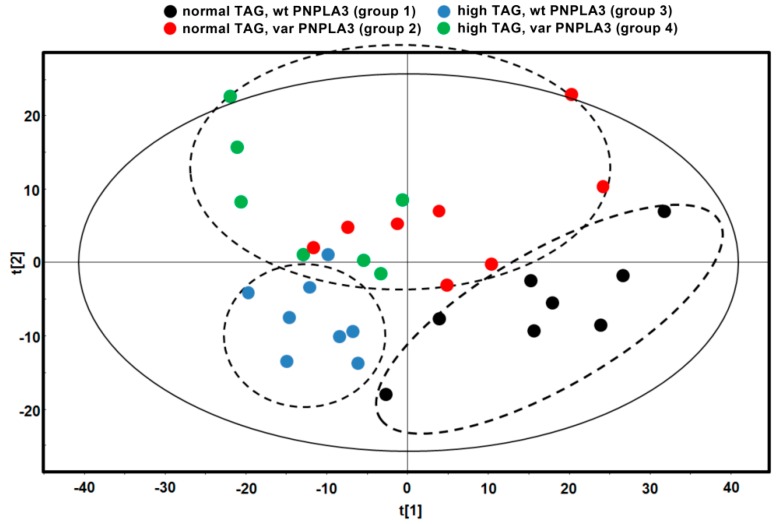
Partial least squares discriminant analysis (PLS-DA) score plot. Each spot represents one liver sample of the denoted group according to component 1 (*x* axis) and 2 (*y* axis). Dashed lines denote possible separation of the groups taking into account all 761 individual lipid species. TAG: liver triacylglycerol content; wt: wild-type allele with I148I; var: I148M variants, which encode I148M (heterozygous) or M148M (homozygous) variants, respectively.

In order to study the effect of I148M PNPLA3 variant on hepatic lipid species in subjects with normal and high liver fat, a complete lipid profile was measured using a novel Lipidyzer approach. The following 13 lipid classes were analyzed: triacylglycerols (TAG), diacylglycerols (DAG), free fatty acids (FFA), ceramides (CER), dihydroceramides (DCER), hexosylceramides (HCER), lactosylceramides (LCER), phosphatidylcholines (PC), lysophosphatidylcholines (LPC), phosphatidylethanolamines (PE), lysophosphatidylethanolamines (LPE), cholesterol esters (CE), and sphingomyelins (SM). Among these lipid classes, 761 individual lipid species were measured and 84 sums of individual classes were calculated. In order to investigate whether the hepatic lipid profile of groups with different PNPLA3 genotype or various liver TAG content was different from each other, we first analyzed the data with multivariate partial least squares discriminant analysis (PLS-DA). PLS-DA showed that normal TAG, wt PNPLA3 (group 1) and high TAG, wt PNPLA3 (group 3) formed distinct groups, however, normal TAG, var PNPLA3 (group 2) and high TAG, var PNPLA3 (group 4) groups were rather similar taking into account all 761 individual lipid species (Figure 1).

### 3.2. I148M PNPLA3 Variant Does Not Change Relative Total Lipid Contents

As a next step, the sum of 13 lipid classes was evaluated. With increased liver TAG content, we observed significantly higher relative levels of TAG and DAG lipids (calculated as % of total lipid content, Table 2). On the other hand, the relative content of FFA, CER, DCER, HCER, PC, LPC, PE, LPE, and SM were significantly lower in high TAG vs. normal TAG groups (Table 2). The presence of I148M PNPLA3 variant did not significantly influence the sum of lipid classes (Table 2).

### 3.3. Increased Liver Fat Content Is Associated with High Proportion of DAG (FA18:1) Species in Subjects with Wild-Type PNPLA3, However, DAG (FA18:1) Remains Unchanged in I148M PNPLA3 Carriers

**Figure 2:**
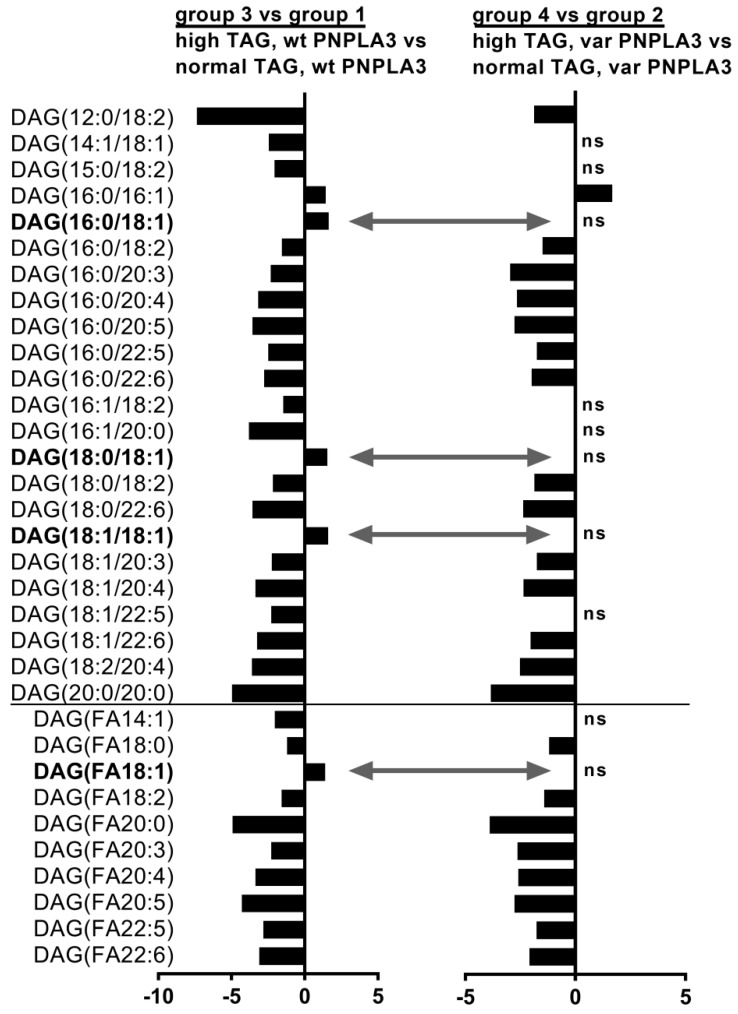
Individual DAGs and their sums, which are significantly changed due to high vs. normal TAG level in subjects with wild-type PNPLA3 (first diagram) and I148M PNPLA3 carriers (second diagram). Column diagrams depict linear fold changes calculated from the proportion of relative individual lipid species and sums, which were significantly altered due to increased liver TAG content in wild-type carriers (first diagram) or in I148M PNPLA3 carriers (second diagram). Positive ratios denote lipids, which are higher in subjects with high liver TAG content compared to normal TAG group, whereas negative ratios denote lipids, which are lower in subjects with high liver TAG content compared to normal TAG group. For DAGs, both fatty acid chains were determined (see as DAG(XX:X/YY:Y). First numbers denote the length of fatty acid chain and second number after “:” denote the number of double bounds. DAG(FAXX:X) depict the sum of DAGs with the denoted fatty acid chain (FA). Bold lipids depict DAG(FA18:1) lipid species, which are significantly increased in wild-type PNPLA3 carriers, but remained unchanged in I148M PNPLA3 carriers. TAG: liver triacylglycerol content; wt: wild-type allele with I148I; var: I148M variants, which encode I148M (heterozygous) or M148M (homozygous) variants, respectively. ns: non-significant differences.

In order to study the combination effect of increased liver fat content and PNPLA3 variant on hepatic lipid profile, we next compared the relative proportion of lipid species in high liver fat to normal liver fat groups from subjects with wild-type (group 3 vs. 1) or with I148M PNPLA3 carriers (group 4 vs. 2) (Figure 2 and Supplementary Table S1).

Although in wild-type carriers many individual DAGs decreased with increased hepatic TAG content, the proportion of DAG (C16:0/C18:1), DAG (C18:0/C18:1), DAG (C18:1/C18:1) as well as the sum of DAG (FA18:1) were increased (Figure 2, first diagram, grey arrows). These FA18:1 containing DAGs remained however, unaltered in I148M PNPLA3 variant carriers (Figure 2, second diagram, grey arrows). Furthermore, various individual CE lipids decreased and many individual shorter TAG lipids were elevated in wild-type subjects due to increased liver TAG content; however, these lipid species remained unaltered in I148M PNPLA3 carriers (Supplementary Table S1). These results indicate that subjects, who carry the I148M PNPLA3 variant do not increase the proportion of DAG (FA18:1) lipid levels upon increased hepatic TAG, although many other lipid species altered similarly compared to wild-type carriers.

## 4. Discussion and Conclusions

The I148M PNPLA3 variant is the best characterized and most influential determinant of NAFLD [19]. Patients with this PNPLA3 variant are also characterized with higher prevalence of NASH and hepatocellular carcinoma [19], however, they show normal insulin sensitivity [8,14]. The underlying mechanism of the dissociation between high liver fat and normal insulin sensitivity remains obscure.

To study the key lipid species, which are known to be involved in hepatic insulin resistance [21], we performed an unbiased lipidomics analysis from subjects with high and normal liver fat content with wild-type or I148M PNPLA3 variants. Our data showed, that DAG (FA18:1) lipid species were elevated in the liver of wild-type carriers upon increased liver fat content, however, these lipids remained unaltered in subjects, who carry I148M PNPLA3 variant. When the enzyme activity of wild-type PNPLA3 was characterized, PNPLA3 (as a lipase) showed hydrolytic activity against mono- (MAG), di- and triacylglycerols [16]. Interestingly, Huang et al. also observed that the wild-type PNPLA3 strongly prefers oleic acid (C18:1)-containing lipids as a substrate [16]. The I148M PNPLA3 variant was not studied for substrate preference, but it showed diminished hydrolytic activity against MAG, DAG, and TAG. Since PNPLA3 owns substrate specificity against C18:1 containing lipids and the I148M variant show diminished TAG hydrolytic activity, it is conceivable that in patients carrying I148M variant, liver TAG(FA18:1) species could be recognized by PNPLA3 in a lower extent than in wild-type carriers, hindering accumulation of DAG (FA18:1). On the other hand, PNPLA3 is not the only lipase metabolizing TAGs and some TAG(FA18:1) lipid species showed lower, but some others showed higher levels in the I148M PNPLA3 variants upon increased liver TAG content when compared to wild-type carriers (Supplementary Table S1).

Rodent studies suggested that PNPLA3 deficiency is associated with reduced hepatic DAG (FA18:1) content. High sucrose diet fed PNPLA3 knock-out mice showed only a decreased DAG (34:1) lipid content (probably consisting of DAG (C16:0/C18:1)), however, all other lipid species (phosphatidic acid, lysophosphatidic acid, TAG or other DAGs) remained unchanged compared to wild-type controls [17]. Knock-down of PNPLA3 with antisense oligonucleotides in high fat diet fed rats resulted in ameliorated hepatic steatosis, which was associated with lower total DAG, DAG (C16:0/C18:1) and DAG (C18:1/C18:1) lipid species [32]. The authors suggested that the lower levels of these DAGs led to reduction of membrane localized (activated) protein kinase C epsilon (PKCε) level, which could not interfere with insulin signaling and this mechanism was postulated to be the reason for the improved hepatic insulin sensitivity found in these PNPLA3 knock-down animals [32]. Jelenik et al. demonstrated that mice with hepatic insulin resistance showed elevated hepatic content of DAG (C16:0/C18:1) and DAG (C18:1/C18:1) lipid species, which was associated with higher PKCε activation and reduced tyrosine phosphorylation of insulin receptor substrate 2 (IRS2), which is a hallmark of impaired insulin signaling [22]. Furthermore, there are several studies [33], which reported an elevated DAG (FA18:1) content in skeletal muscle of insulin resistant patients with obesity [34] and type 2 diabetes [35]. The authors claimed that the elevated DAG levels could activate PKC theta (PKCΘ) in skeletal muscle, which in turn leads to impaired insulin signaling causing insulin resistance [35]. Moreover, elevated DAG species were also found in the liver of subjects, who showed hepatic insulin resistance [23]. From all DAG species, hepatic cytosolic level of DAG (C16:0/C18:1) and DAG (C18:1/C18:1) were two out of the three most abundant DAGs, which showed the strongest negative correlation with suppression of endogenous glucose production (EGP) [23]. Impairment in the suppression of EGP is a sign for hepatic insulin resistance [36,37]. These results indicate that elevation of DAG (FA18:1) lipid species is a characteristic of insulin resistance and impairment in PNPLA3 function is associated with lower content of hepatic DAG (FA18:1). Whether DAG (FA18:1) is attributed to specific functions in comparison with other DAG species is not clarified yet, but it is possible. Dziewulska et al. reported that mice fed with triolein diet (TAG (C18:1/C18:1/C18:1)) resulted in elevation of DAG (FA18:1) in skeletal muscle, which was associated with higher PKCΘ activation but lower serine phosphorylation of protein kinase B and diminished glucose transporter 4 translocation [38], which are signs for insulin resistance [39]. The authors also observed, that tristearin diet (TAG (C18:0/C18:0/C18:0)) feeding did not exert the former effects [38]. These results suggest that DAG (FA18:1) possibly owns a specific function among DAGs and it could serve as a strong activator of PKCs (PKCε in the liver and PKCΘ in the muscle), which, in turn, could diminish insulin signaling.

The PNPLA3 variant cohorts consisted of mainly heterozygous (I148M) and only one homozygous (M148M) carrier in each group. Previous studies also combined hetero- and homozygous carriers of the PNPLA3 variant and did not report differences in lipid composition or insulin sensitivity [8,10,15,40] suggesting that these variants are comparable. We, therefore, also analyzed all carriers of the PNPLA3 I148M/M148M genotype together in this study. The limitations of this study are the comorbidities of the subjects and the small study size, however, surgical samples were necessary to obtain sufficient tissue for the lipid analyses. Since we did not have detailed information on alcohol intake of the patients, it cannot be ruled out that some of the patients had an alcohol related cause of fatty liver rather than NAFLD. Furthermore, liver samples fulfilling the criteria of the defined groups were very limited. Therefore, further sub-analyses exceeding the initially selected groups are not possible in this study.

**Figure 3:**
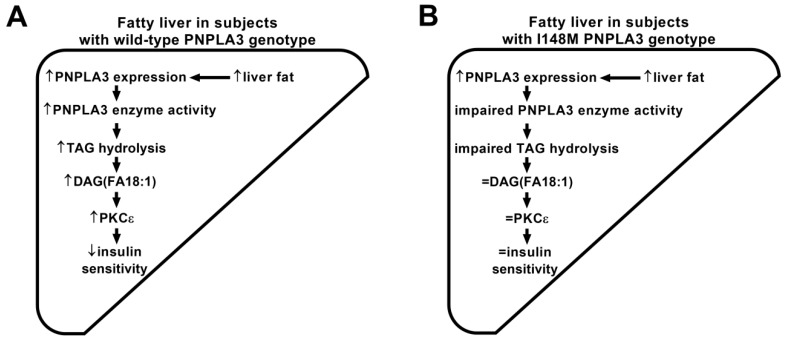
Hypothetical scheme showing the association between I148M PNPLA3 variant and normal insulin sensitivity. Arrows demonstrate higher (↑) or lower (↓) metabolite contents, transcript levels, enzyme activities or insulin sensitivity, respectively. Previous data shown, that liver fat content is positively associated with hepatic mRNA expression of PNPLA3, which was not altered in subjects carrying I148M PNPLA3 variant [10,14,32]. Our lipid data showed that hepatic DAG (FA18:1) species were elevated in fatty liver of wild-type PNPLA3 carriers (**A**), which was not observed in I148M PNPLA3 carriers (**B**). Elevated DAG (FA18:1) in the liver was shown to activate protein kinase c epsilon (PKCε), which, in turn, reduces tyrosin phosphorylation of insulin receptor substrate 2 (P-Tyr-IRS2) [22,23], a key molecule transmitting insulin signaling in the liver [39]. Due to the attenuated tyrosin phosphorylation of IRS2, insulin sensitivity could be impaired (as postulated earlier [22,23]) in subjects carrying wild-type PNPLA3 allele, but not in I148M PNPLA3 carriers.

We found that fatty liver in subjects carrying wild-type PNPLA3 is associated with elevated hepatic DAG (FA18:1) content. These DAG (FA18:1) species were shown to disturb insulin signaling in the liver [22,23]. However, hepatic DAG (FA18:1) species remained unaltered in subjects carrying I148M PNPLA3 allele with fatty liver. Therefore, we hypothesize that I148M PNPLA3 carriers may be protected from insulin resistance via the unaltered content of DAG (FA18:1) species due to impaired PNPLA3 TAG lipase activity (Figure 3).

## Figures and Tables

**Table 1:** Characteristics of study groups.

Characteristic	Group 1 Normal TAG, wt PNPLA3	Group 2 Normal TAG, var PNPLA3	Group 3 High TAG, wt PNPLA3	Group 4 High TAG, var PNPLA3
Age (years)	59.3 ± 12.6	60.6 ± 16.4	64.0 ± 11.8	65.1 ± 14.5
Body weight (kg)	79.3 ± 9.9	80.3 ± 13.5	86.2 ± 11.1	87.1 ± 13.1
BMI (kg/m^2^)	26.1 ± 3.2	28.0 ± 5.3	31.0 ± 3.4	28.6 ± 4.0
ALT (U/L)	24.5 ± 6.5	22.0 ± 5.2	31.1 ± 9.3	35.7 ± 18.6
Liver fat (%)	1.1 ± 0.8	1.5 ± 0.7	5.9 ± 2.0 ***	7.6 ± 2.9 ***
PNPLA3 ^148^ (II/IM/MM) (*n*)	8/0/0	0/7/1	8/0/0	0/6/1
Sex (m/f) (*n*)	6/2	4/4	5/3	5/2
Number of subjects (*n*)	8	8	8	7

TAG: liver triacylglycerol content; PNPLA3: patatin-like phospholipase domain-containing protein 3; wt: wild-type allele with I148I; var: I148M variants, which encode I148M (heterozygous) or M148M (homozygous) variants, respectively. ALT: alanine aminotransferase, BMI: body mass index. Numbers denote averages ± standard deviations in the first five lines. *** denotes significant differences between group 3 vs. 1 or group 4 vs. 2 illustrating the effect of liver TAG content; *p* < 0.001. Significance was calculated using ANOVA with Holm-Sidak’s post-hoc test and assumed as *p* < 0.05. By the comparisons of group 2 vs. 1 and group 4 vs. 3 no significant differences were found for I148M PNPLA3 variant vs. wild-type carriers.

**Table 2:** Relative lipid contents of individual classes in percent.

Lipid Class %	Group 1 Normal TAG, wt PNPLA3	Group 2 Normal TAG, var PNPLA3	Group 3 High TAG, wt PNPLA3	Group 4 High TAG, var PNPLA3
TAG	25.63 ± 12.49	35.22 ± 15.30	68.64 ± 11.26 ***	69.82 ± 11.86 ***
DAG	0.60 ± 0.30	0.57 ± 0.17	0.92 ± 0.12 *	1.02 ± 0.20 **
FFA	7.23 ± 3.57	6.07 ± 1.71	2.60 ± 1.12 ***	2.37 ± 1.01 **
CER	0.26 ± 0.04	0.25 ± 0.08	0.10 ± 0.03 ***	0.10 ± 0.05 ***
DCER	0.03 ± 0.01	0.03 ± 0.01	0.01 ± 0.00 ***	0.01 ± 0.01 ***
HCER	0.07 ± 0.02	0.05 ± 0.02	0.03 ± 0.01 ***	0.02 ± 0.01 **
LCER	0.06 ± 0.01	0.06 ± 0.03	0.04 ± 0.02	0.03 ± 0.01
PC	38.51 ± 9.74	33.06 ± 8.63	14.86 ± 6.41 ***	14.75 ± 7.83 ***
LPC	0.66 ± 0.22	0.60 ± 0.14	0.24 ± 0.14 ***	0.28 ± 0.17 **
PE	20.43 ± 5.80	17.84 ± 5.82	8.19 ± 3.24 ***	7.29 ± 3.10 ***
LPE	0.16 ± 0.05	0.15 ± 0.03	0.06 ± 0.03 ***	0.07 ± 0.04 ***
CE	2.45 ± 0.34	2.84 ± 0.45	2.73 ± 0.60	2.86 ± 0.48
SM	3.91 ± 0.81	3.28 ± 0.81	1.59 ± 0.67 ***	1.37 ± 0.60 ***

TAG: triacylglycerols, DAG: diacylglycerols, FFA: free fatty acids, CER: ceramides, DCER: dihydroceramides, HCER: hexosylceramides, LCER: lactosylceramides, PC: phosphatidylcholines, LPC: lysophosphatidylcholines, PE: phosphatidylethanolamines, LPE: lysophosphatidylethanolamines, CE: cholesterol esters, SM: sphingomyelins; wt: wild-type allele with I148I; var: I148M variants, which encode I148M (heterozygous) or M148M (homozygous) variants, respectively. Numbers denote averages ± standard deviations. * denotes significant differences between group 3 vs. 1 or group 4 vs. 2 illustrating the effect of liver TAG content; * *p* < 0.05, ** *p* < 0.01, *** *p* < 0.001. Significance was calculated using ANOVA with Holm-Sidak´s post-hoc test and assumed as *p* < 0.05. By the comparisons of group 2 vs. 1 and group 4 vs. 3 no significant differences were found for I148M PNPLA3 variant vs. wild-type carriers.

## Supplementary Materials

Supplementary materials can be found at http://www.mdpi.com/2072-6643/10/9/1314/s1. Table S1. Lipids and sum of lipids, which are significantly changed due to high vs. normal TAG level in subjects.

## Abbreviations

DAG	Diacylglycerol
FA	Fatty acid
group 1	Subjects with normal liver fat and wild-type PNPLA3
group 2	Subjects with normal liver fat and I148M/M148M PNPLA3 variants
group 3	Subjects with high TAG and wild-type PNPLA3
group 4	Subjects with high TAG and I148M/M148M PNPLA3 variants
MAG	Monoacylglycerol
NAFLD	Nonalcoholic fatty liver disease
NASH	Nonalcoholic steatohepatitis
PKC	Protein kinase C
PNPLA3	Patatin-like phospholipase domain-containing protein 3
TAG	Triacylglycerol
var	I148M/M148M PNPLA3 variants
wt	Wild-type PNPLA3 allele

## References

[1] Stefan N., Schick F., Häring H.U. (2017). Causes, characteristics, and consequences of metabolically unhealthy normal weight in humans. Cell Metab..

[2] Stefan N., Häring H.U. (2013). The role of hepatokines in metabolism. Nat. Rev. Endocrinol..

[3] Younossi Z., Anstee Q.M., Marietti M., Hardy T., Henry L., Eslam M., George J., Bugianesi E. (2018). Global burden of NAFLD and NASH: Trends, predictions, risk factors and prevention. Nat. Rev. Gastroenterol. Hepatol..

[4] Younossi Z.M., Loomba R., Rinella M.E., Bugianesi E., Marchesini G., Neuschwander-Tetri B.A., Serfaty L., Negro F., Caldwell S.H., Ratziu V. (2018). Current and future therapeutic regimens for nonalcoholic fatty liver disease and nonalcoholic steatohepatitis. Hepatology.

[5] Böhm A., Hoffmann C., Irmler M., Schneeweiss P., Schnauder G., Sailer C., Schmid V., Hudemann J., Machann J., Schick F. (2016). TGF-beta contributes to impaired exercise response by suppression of mitochondrial key regulators in skeletal muscle. Diabetes.

[6] Franko A., Neschen S., Rozman J., Rathkolb B., Aichler M., Feuchtinger A., Brachthäuser L., Neff F., Kovarova M., Wolf E. (2017). Bezafibrate ameliorates diabetes via reduced steatosis and improved hepatic insulin sensitivity in diabetic TallyHo mice. Mol. Metab..

[7] Goto T., Itoh M., Suganami T., Kanai S., Shirakawa I., Sakai T., Asakawa M., Yoneyama T., Kai T., Ogawa Y. (2018). Obeticholic acid protects against hepatocyte death and liver fibrosis in a murine model of nonalcoholic steatohepatitis. Sci. Rep..

[8] Romeo S., Kozlitina J., Xing C., Pertsemlidis A., Cox D., Pennacchio L.A., Boerwinkle E., Cohen J.C., Hobbs H.H. (2008). Genetic variation in PNPLA3 confers susceptibility to nonalcoholic fatty liver disease. Nat. Genet..

[9] Baclig M.O., Lozano-Kuhne J.P., Mapua C.A., Gopez-Cervantes J., Natividad F.F. (2014). Genetic variation I148M in patatin-like phospholipase 3 gene and risk of non-alcoholic fatty liver disease among Filipinos. Int. J. Clin. Exp. Med..

[10] Kantartzis K., Peter A., Machicao F., Machann J., Wagner S., Konigsrainer I., Konigsrainer A., Schick F., Fritsche A., Häring H.U. (2009). Dissociation between fatty liver and insulin resistance in humans carrying a variant of the patatin-like phospholipase 3 gene. Diabetes.

[11] Stojkovic I.A., Ericson U., Rukh G., Riddestrale M., Romeo S., Orho-Melander M. (2014). The PNPLA3 Ile148Met interacts with overweight and dietary intakes on fasting triglyceride levels. Genes Nutr..

[12] Sevastianova K., Santos A., Kotronen A., Hakkarainen A., Makkonen J., Silander K., Peltonen M., Romeo S., Lundbom J., Lundbom N. (2012). Effect of short-term carbohydrate overfeeding and long-term weight loss on liver fat in overweight humans. Am. J. Clin. Nutr..

[13] Scorletti E., West A.L., Bhatia L., Hoile S.P., McCormick K.G., Burdge G.C., Lillycrop K.A., Clough G.F., Calder P.C., Byrne C.D. (2015). Treating liver fat and serum triglyceride levels in NAFLD, effects of PNPLA3 and TM6SF2 genotypes: Results from the WELCOME trial. J. Hepatol..

[14] Peter A., Kovarova M., Nadalin S., Cermak T., Konigsrainer A., Machicao F., Stefan N., Häring H.U., Schleicher E. (2014). PNPLA3 variant I148M is associated with altered hepatic lipid composition in humans. Diabetologia.

[15] Luukkonen P.K., Zhou Y., Sadevirta S., Leivonen M., Arola J., Oresic M., Hyotylainen T., Yki-Jarvinen H. (2016). Hepatic ceramides dissociate steatosis and insulin resistance in patients with non-alcoholic fatty liver disease. J. Hepatol..

[16] Huang Y., Cohen J.C., Hobbs H.H. (2011). Expression and characterization of a PNPLA3 protein isoform (I148M) associated with nonalcoholic fatty liver disease. J. Biol. Chem..

[17] Kumari M., Schoiswohl G., Chitraju C., Paar M., Cornaciu I., Rangrez A.Y., Wongsiriroj N., Nagy H.M., Ivanova P.T., Scott S.A. (2012). Adiponutrin functions as a nutritionally regulated lysophosphatidic acid acyltransferase. Cell Metab..

[18] Mancina R.M., Matikainen N., Maglio C., Soderlund S., Lundbom N., Hakkarainen A., Rametta R., Mozzi E., Fargion S., Valenti L. (2015). Paradoxical dissociation between hepatic fat content and de novo lipogenesis due to PNPLA3 sequence variant. J. Clin. Endocrinol. Metab..

[19] Petaja E.M., Yki-Jarvinen H. (2016). Definitions of normal liver fat and the association of insulin sensitivity with acquired and genetic NAFLD-A systematic review. Int. J. Mol. Sci..

[20] Li J.Z., Huang Y., Karaman R., Ivanova P.T., Brown H.A., Roddy T., Castro-Perez J., Cohen J.C., Hobbs H.H. (2012). Chronic overexpression of PNPLA3I148M in mouse liver causes hepatic steatosis. J. Clin. Investig..

[21] Samuel V.T., Shulman G.I. (2018). Nonalcoholic fatty liver disease as a nexus of metabolic and hepatic diseases. Cell Metab..

[22] Jelenik T., Kaul K., Sequaris G., Flogel U., Phielix E., Kotzka J., Knebel B., Fahlbusch P., Horbelt T., Lehr S. (2017). Mechanisms of insulin resistance in primary and secondary nonalcoholic fatty liver. Diabetes.

[23] Ter Horst K.W., Gilijamse P.W., Versteeg R.I., Ackermans M.T., Nederveen A.J., la Fleur S.E., Romijn J.A., Nieuwdorp M., Zhang D., Samuel V.T. (2017). Hepatic diacylglycerol-associated protein kinase cepsilon translocation links hepatic steatosis to hepatic insulin resistance in humans. Cell Rep..

[24] Ollero M. (2004). Methods for the study of lipid metabolites in cystic fibrosis. J. Cyst. Fibros..

[25] Lehmann R., Franken H., Dammeier S., Rosenbaum L., Kantartzis K., Peter A., Zell A., Adam P., Li J., Xu G. (2013). Circulating lysophosphatidylcholines are markers of a metabolically benign nonalcoholic fatty liver. Diabetes Care.

[26] Franko A., Huypens P., Neschen S., Irmler M., Rozman J., Rathkolb B., Neff F., Prehn C., Dubois G., Baumann M. (2016). Bezafibrate improves insulin sensitivity and metabolic flexibility in STZ-induced diabetic mice. Diabetes.

[27] Markgraf D.F., Al-Hasani H., Lehr S. (2016). Lipidomics-reshaping the analysis and perception of type 2 diabetes. Int. J. Mol. Sci..

[28] Ubhi B.K. (2018). Direct Infusion-Tandem Mass Spectrometry (DI-MS/MS) analysis of complex lipids in human plasma and serum using the lipidyzer platform. Methods Mol. Biol..

[29] Peter A., Kantartzis K., Machicao F., Machann J., Wagner S., Templin S., Königsrainer I., Königsrainer A., Schick F., Fritsche A. (2012). Visceral obesity modulates the impact of apolipoprotein C3 gene variants on liver fat content. Int. J. Obes..

[30] Franko A., Kovarova M., Feil S., Feil R., Wagner R., Heni M., Köngisrainer A., Ruoß M., Nüssler A.K., Weigert C. (2018). cGMP-dependent protein kinase I (cGKI) modulates human hepatic stellate cell activation. Metabolism.

[31] Chen S., Hoene M., Li J., Li Y., Zhao X., Häring H.U., Schleicher E.D., Weigert C., Xu G., Lehmann R. (2013). Simultaneous extraction of metabolome and lipidome with methyl tert-butyl ether from a single small tissue sample for ultra-high performance liquid chromatography/mass spectrometry. J. Chromatogr. A.

[32] Kumashiro N., Yoshimura T., Cantley J.L., Majumdar S.K., Guebre-Egziabher F., Kursawe R., Vatner D.F., Fat I., Kahn M., Erion D.M. (2013). Role of patatin-like phospholipase domain-containing 3 on lipid-induced hepatic steatosis and insulin resistance in rats. Hepatology.

[33] Amati F. (2012). Revisiting the diacylglycerol-induced insulin resistance hypothesis. Obes. Rev..

[34] Moro C., Galgani J.E., Luu L., Pasarica M., Mairal A., Bajpeyi S., Schmitz G., Langin D., Liebisch G., Smith S.R. (2009). Influence of gender, obesity, and muscle lipase activity on intramyocellular lipids in sedentary individuals. J. Clin. Endocrinol. Metab..

[35] Szendroedi J., Yoshimura T., Phielix E., Koliaki C., Marcucci M., Zhang D., Jelenik T., Muller J., Herder C., Nowotny P. (2014). Role of diacylglycerol activation of PKCtheta in lipid-induced muscle insulin resistance in humans. Proc. Natl. Acad. Sci. USA.

[36] Franko A., von Kleist-Retzow J.C., Neschen S., Wu M., Schommers P., Böse M., Kunze A., Hartmann U., Sanchez-Lasheras C., Stoehr O. (2014). Liver adapts mitochondrial function to insulin resistant and diabetic states in mice. J. Hepatol..

[37] Ayala J.E., Bracy D.P., McGuinness O.P., Wasserman D.H. (2006). Considerations in the design of hyperinsulinemic-euglycemic clamps in the conscious mouse. Diabetes.

[38] Dziewulska A., Dobrzyn P., Jazurek M., Pyrkowska A., Ntambi J.M., Dobrzyn A. (2012). Monounsaturated fatty acids are required for membrane translocation of protein kinase C-theta induced by lipid overload in skeletal muscle. Mol. Membr. Biol..

[39] Boucher J., Kleinridders A., Kahn C.R. (2014). Insulin receptor signaling in normal and insulin-resistant states. Cold Spring Harb. Perspect. Biol..

[40] Hyysalo J., Gopalacharyulu P., Bian H., Hyotylainen T., Leivonen M., Jaser N., Juuti A., Honka M.J., Nuutila P., Olkkonen V.M. (2014). Circulating triacylglycerol signatures in nonalcoholic fatty liver disease associated with the I148M variant in PNPLA3 and with obesity. Diabetes.

